# The composting microbiome and a multifunctional *Bacillus tequilensis* JZF3 with straw degradation and pathogen inhibition

**DOI:** 10.3389/fmicb.2026.1768200

**Published:** 2026-03-23

**Authors:** Ruiyao Liu, Jing Liu, Zhe Li, Ziyan Wang, Jilin Ding, Jinfeng Yuan, Bo Yao, Xiangli Dong, Wendian Dai, Zhengquan Huang, Cheng-Sheng Zhang, Wenjian Zhang, Yanfen Zheng

**Affiliations:** 1 Tobacco Research Institute of Chinese Academy of Agricultural Sciences, Qingdao, China; 2 Zunyi Branch of Guizhou Tobacco Company, Zunyi, China; 3 Zunyi Daxing Compound Fertilizer Co., Ltd., Zunyi, China; 4 Zunyi Maxing Organic Fertilizer Co., Ltd., Zunyi, China; 5 Qingdao Center of Technology Innovation for Microbial Germplasm, Key Laboratory of Bio-resources Evaluation and Utilization in Saline-Alkali Soil, Qingdao, China

**Keywords:** aerobic composting, *Bacillus tequilensis*, biological control, lignocellulose degradation, microbial community

## Abstract

Aerobic composting is a sustainable approach for converting organic waste into bio-fertilizer, where microorganisms play a central role in the degradation of recalcitrant lignocellulose. This study employed high-throughput sequencing to analyze the dynamic changes in bacterial and fungal communities during composting using distillers’ grains, oil cake and cattle manure as raw materials. The results revealed pronounced successional changes in both microbial community structure and predicted function over time. Specifically, Firmicutes and Ascomycota were the dominant bacterial and fungal phyla, respectively, with the genus *Bacillus* maintaining high abundance throughout the process. The predicted functional profile indicated a shift in bacterial functions from initial xenobiotic biodegradation to core metabolic processes (such as energy and carbohydrate metabolism) in later stages. A total of 97 bacterial strains belonging to 38 species were isolated from different composting samples, with four strains (*Bacillus licheniformis* JZF8, *B. altitudinis* JZF2, *B. tequilensis* JZF3, and *B. siamensis* FJ3-3) showing strong cellulase, ligninase and protease activities. Among them, strain JZF3 not only exhibited these enzymatic activities and significant antagonistic activity against plant pathogens, but also was a dominant culturable species within the compost community. Furthermore, strain JZF3 was able to directly degrade rice straw without chemical pretreatment, achieving a degradation rate of 22.5%. The resulting degradation products also significantly promoted the growth of tobacco seedlings. This study identifies *B. tequilensis* JZF3 as a multifunctional agent that combines straw degradation with pathogen suppression and plant growth promotion, offering a novel strategy for synergizing agricultural waste recycling with disease control.

## 1 Introduction

Aerobic composting is a microbe-mediated aerobic and thermophilic solid-state fermentation process that converts organic waste into bio-fertilizers for crop growth (Huang et al., 2022; Hawkesford et al., 2012). The complete composting process includes the heating, thermophilic, cooling and maturation stages (Du et al., 2020; Chin et al., 2020). During this process, microorganisms facilitate the mineralization and humification of the compost material through fermentation, ultimately converting it into organic fertilizer (Alengebawy et al., 2024). High-quality organic fertilizers can improve soil quality, enhance nutrient availability of nutrients, and increase crop yield (Edmeades, 2003). Thus, aerobic composting serves as both an effective method for waste resource utilization and an important way for enhancing soil fertility.

Organic fertilizer raw materials, such as distiller’s grains, oil cakes and cattle manure, are usually rich in lignocellulose, which is composed of cellulose, hemicellulose and lignin (Mori et al., 2015). These components have stable structures and are difficult to degrade. In particular, lignin is embedded between cellulose and hemicellulose, forming a complex heterogeneous network and becoming the rate-limiting factor for aerobic composting (Usmani et al., 2020). Numerous studies have shown that the degradation of lignocellulose in compost mainly occurs during the thermophilic phase (Ma et al., 2020), where bacteria (especially *Bacillus*) and actinomycetes are the main microbial groups (Guo et al., 2021; Yang et al., 2022; Li et al., 2020; Duan et al., 2020). In the later stage of composting, some microorganisms can further degrade recalcitrant substances such as lignin (Bohacz, 2017) and promote compost maturation. Therefore, investigating the microbial community composition is pivotal for understanding the microbial mechanisms of organic matter transformation during composting (Finore et al., 2023; Li et al., 2023).

In addition to low soil fertility in tobacco monoculture systems, diseases (e.g., black shank and brown spot diseases) severely constrain tobacco yield (Bai et al., 2024; Hou et al., 2016). Exploring microbial agents that enhance composting while simultaneously suppressing plant disease offers a promising strategy to address these challenges. Previous studies have reported the dynamic changes in microbial communities during composting (Wei et al., 2018; Akyol et al., 2019), and several strains with lignin degradation capabilities have been isolated from different habitats such as soil, decayed wood (Taylor et al., 2012), and animal intestines (Dumond et al., 2021). For instance, *Bacillus amyloliquefaciens* can degrade lignin at rates of 28.55–46.7% (Mei et al., 2020). *Bacillus flexus, Bacillus lignophilus,* and *Bacillus sonorensis* also have lignin degradation capabilities, with degradation rates reaching 30, 9.1, and 7.68%, respectively, (Kumar et al., 2019; Zhu et al., 2017; Wang et al., 2021). However, existing studies have mostly focused on strains with single function, and microorganisms that combine straw degradation, pathogen inhibition, and plant growth promotion capabilities remain rarely reported.

In this study, composting was conducted using distiller’s grains, oil cake and cattle manure as raw materials. We aimed to: (i) analyze shifts in microbial composition across different fermentation stages using high-throughput sequencing; (ii) isolate culturable bacteria and screen them for the production of key enzymes (cellulase, ligninase, protease) as well as for their antagonistic activity against phytopathogens; and (iii) evaluate the effect of the microbial degradation products on tobacco growth promotion. The findings are expected to provide novel microbial resources capable of simultaneously improving aerobic composting efficiency and suppressing pathogen inhibition, thereby offering a multi-functional strategy for sustainable agricultural waste management.

## 2 Materials and methods

### 2.1 Sample collection

Raw materials including 40% distillers’ grains, 40% microbial residue, and 20% cattle manure were used for composting. The composting pile was constructed with dimensions of approximately 40 m × 12 m × 1.8 m (length × width × height). The initial carbon-to-nitrogen (C/N) ratio was adjusted to approximately 28:1, and the moisture content was maintained at approximately 60%. No mechanical turning was performed during the monitored period. Samples were collected on days 0, 3, 6, 12 and 18 of composting, each with six biological replicates. Each compost sample was subdivided into two portions: one was stored at −80 °C for DNA sequencing, and the other was designated for microbial isolation and cultivation.

### 2.2 DNA extraction and PCR amplification

Genomic DNA from all samples was extracted using the FastDNA® Spin Kit for Soil (MP Biomedicals), and the extracted genomic DNA was detected by 1% agarose gel electrophoresis. Six independent biological replicates per time point were sequenced individually. The V3-V4 region of the bacterial 16S rRNA gene was amplified by PCR using primers 515F (5’-CCTAYGGGRBGCASCAG-3′) and 806R (5’-GGACTACNNGGGTATCTAAT-3′). The ITS1 region of the fungal ITS gene was amplified using primers ITS1F (5’-CTTGGTCATTTAGAGGAAGTAA −3′) and ITS2R (5’-GCTGCGTTCTTCATCGATGC-3′). All PCR reactions were carried out with 15 μL of Phusion High-Fidelity PCR Master Mix; 0.2 μM of forward and reverse primers, and about 10 ng template DNA. Thermal cycling consisted of initial denaturation at 98 °C for 1 min, followed by 30 cycles of denaturation at 98 °C for 10 s, annealing at 50 °C for 30 s, and elongation at 72 °C for 30 s, with a final extension at 72 °C for 5 min. The PCR products were purified using magnetic bead purification. Equimolar amounts of PCR products from different samples were pooled based on their concentrations. A sequencing library was then prepared, and sequencing was performed on the NovaSeq PE250 platform.

### 2.3 High-throughput sequencing data processing

The QIIME2 software (Bolyen et al., 2019) was used for quality control of the sequencing data and subsequent analysis. The obtained raw reads were spliced and filtered to obtain valid data, and then denoising was performed with the QIIME2 to obtain Amplicon Sequence Variants (ASVs) (Callahan et al., 2016). The Silva 138.1 database and the Unite v9.0 database were used for bacterial and fungal species annotation, respectively. Principal Coordinate Analysis (PCoA) was performed to visualize principal coordinates of the microbial community using the ade4 and ggplot2 in R software (Version 4.0.3). Bacterial community function was predicted using Tax4Fun. For fungal communities, the FunGuild tool was used to classify trophic modes and analyze specific functions.

### 2.4 Bacterial isolation and identification

The fermentation product was thoroughly mixed with sterile water in a sterile 50 mL centrifuge tube by vigorous shaking. Serial dilution method was used to obtain dilutions of different concentrations (Wang Y. et al., 2020). Then, 100 μL of 10^−1^, 10^−2^, 10^−3^ dilutions were spread on Tryptic Soy Agar medium (TSA; 10.0 g/L Tryptone, 3.0 g/L Beef Extract, 5.0 g/L NaCl, 20.0 g/L Agar) and incubated at 28 °C for 2–3 days. Single colonies were picked and streaked onto fresh TSA plates for purification. Genomic DNA was extracted from each purified isolate, and the 16S rRNA gene was amplified using primers 27F (5’-AGAGTTTGATCCTGGCTCAG-3′) and 1492R (5’-CTACGGCTACCTTGTTACGA-3′). PCR amplification was performed in a 25 μL mixture containing 12.5 μL of 2 × Taq Master Mix, 1 μL of each primer, 10 μL ddH_2_O and 0.5 μL genomic DNA. The PCR conditions were as follows: pre-denaturation at 95 °C for 5 min; 35 cycles of denaturation at 95 °C for 1 min, annealing at 55 °C for 1 min, extension at 72 °C for 1.5 min; and a final extension at 72 °C for 10.0 min. The amplified products were sequenced by BGI Co., Ltd. (Beijing, China). The resulting sequences were submitted to EzBioCloud^1^ for bacterial identification. MEGA 11.0 software was used to perform multiple sequence alignment with CLUSTALX. The resulting tree was visualized with iTOL^2^ (Letunić and Bork, 2024).

### 2.5 Screening of enzyme-producing strains

#### 2.5.1 Screening of cellulose-degrading strains

The cellulose degradation capacity of the isolated strains was assessed using the Congo red staining method (Teather and Wood, 1982). The isolated bacteria were inoculated into Tryptic Soy Broth medium (TSB; 10.0 g/L Tryptone, 3.0 g/L Beef Extract, 5.0 g/L NaCl) and incubated at 28 °C with shaking at 180 rpm for 24 h. Subsequently, each strain was spotted onto carboxymethyl cellulose (CMC) medium (10 g/L CMC-Na, 1 g/L K_2_HPO_4_, 1.0 g/L NH_4_NO_3_, 0.02 g/L CaCl_2_, 0.2 g/L MgSO_4_·7H_2_O, 0.05 g/L FeCl_3_· 6H_2_O) with three replicates and incubated at 28 °C for 3 days. The positive control strain (a known enzyme-producing strain) (Yadav and Dubey, 2018) and negative control (medium only) were included. Afterwards, the plates were stained with 3 mL of Congo red solution (1 g/L) for 45 min at room temperature and then destained with 1 mol/L NaCl for 45 min. The diameter of colony d (cm) and hydrolysis zone D (cm) were measured using a digital Vernier caliper (Rath et al., 2025), and the cellulose-degrading capacity of the strains was evaluated based on the ratio of the hydrolysis zone diameter to the bacterial colony diameter (D/d) (Javaheri-Kermani and Asoodeh, 2019).

#### 2.5.2 Screening of lignin-degrading strains

The lignin degradation capacity of the isolated strains was screened by the aniline blue plate decolorization method (Archibald, 1992). The isolated bacteria were inoculated into TSB medium and incubated at 28 °C with shaking at 180 rpm for 24 h. Subsequently, each strain was spotted onto Potato Dextrose Agar (PDA) supplemented with Aniline Blue medium (46.0 g/L Potato Dextrose Agar, 0.10 g/L Aniline Blue) with three replicates and incubated at 28 °C for 3 days. The positive control strain (a known enzyme-producing strain) (Kumar et al., 2019) and negative control (medium only) were included. The diameter of colony d (cm) and hydrolysis zone D (cm) were measured using a digital Vernier caliper (Rath et al., 2025), and the lignin-degrading capacity of the strains was evaluated based on the ratio of the hydrolysis zone diameter to the bacterial colony diameter (D/d).

#### 2.5.3 Screening of protein-degrading strains

The protein degradation capacity of the isolated strains was screened using casein medium (35 g/L Casein Agar) and skim milk medium (20 g/L Skim Milk, Peptone 5 g/L, Yeast Extract 2 g/L, NaCl 2 g/L, Agar 15 g/L). Protease produced by strains can decompose proteins in the medium, resulting in transparent hydrolysis rings (Gerhardt et al., 1994). The isolated bacteria were inoculated into TSB medium and incubated at 28 °C with shaking at 180 rpm for 24 h. Subsequently, each strain was spotted onto casein medium and skim milk medium with three replicates per strain and incubated at 28 °C for 3 days. The positive control strain (a known enzyme-producing strain) (Ma et al., 2024) and negative control (medium only) were included. The diameter of colony d (cm) and hydrolysis zone D (cm) were measured using a digital Vernier caliper (Rath et al., 2025), and the protein-degrading capacity of the strains was evaluated based on the ratio of the hydrolysis zone diameter to the bacterial colony diameter (D/d).

### 2.6 Antagonistic effects of the strains

The ability of the strains to inhibit *Phytophthora parasitica* and *Alternaria alternata* was determined by the plate confrontation method (Pliego et al., 2011). *P. parasitica* and *A. alternata* were inoculated onto oatmeal agar (OA) medium and cultured for 3 days. The bacterial strain was inoculated into TSB medium and incubated at 28 °C with shaking at 180 rpm for 24 h. The bacterial suspension was adjusted to OD_600_ = 1.0. Mycelial plugs (5 mm) taken from the edge of fungal colonies were placed at the center of fresh OA medium. 3 μL of bacterial suspension was spotted 2.5 cm away from the fungal plug. Three replicates were prepared for each treatment, while control plates did not receive bacterial suspension. Inhibition rate (%) = [(Dc − Dt) / Dc] × 100, where Dc and Dt represent the mycelial diameter in the control and treatment groups, respectively.

### 2.7 Determination of straw degradation and growth-promoting capacity of strain JZF3

The degradation rate of straw by strain JZF3 was determined by the weight loss method (Zhang et al., 2022; Wang Z. Y. et al., 2020). Strain JZF3 was inoculated into TSB medium and incubated at 28 °C with shaking at 180 rpm overnight. Bacterial cells were harvested (OD_600_ = 1.0) and washed with sterile water for three times. Then, they were inoculated into liquid culture medium with sterilized rice straw (ground and sieved to a particle size of ≤ 2 mm) as the sole carbon source (25 g/L Rice Straw Powder, 1.0 g/L KH_2_PO_4_, 0.2 g/L MgSO_4_, 1.0 g/L K_2_HPO_4_, 2.0 g/L (NH_4_)_2_SO_4_, and 0.1 g/L CaCl_2_). A medium without bacterial inoculation was used as the control to account for potential non-biological mass loss. All cultures were incubated at 28 °C with shaking at 180 rpm for 7 days. The residual straw was dried to a constant weight, and the degradation rate was calculated based on the mass loss compared to a non-inoculated control. Degradation rate (%) = (W_0_-W_1_) / W_0_ × 100%, where W_0_ represents the drying mass (g) of the remaining straw in the control group, W_1_ represents the drying mass (g) of the remaining straw in the treatment group. The experiment was conducted with three biological replicates.

To assess the plant-growth-promoting potential of the metabolites produced during straw degradation, a pot experiment was conducted using tobacco seedlings. The treatment group received a 7-fold diluted supernatant of JZF3-fermented rice straw, while the control received an equal volume of uninoculated supernatant. Each pot was supplied with 20 mL of supernatant, and the seedlings were cultivated for 10 days. After the growth period, tobacco seedling fresh weight, root length and leaf area (calculated as 0.6345 × leaf length × leaf width) were measured. The pot assay included three replicates per treatment, with 10 seedlings in each replicate.

## 3 Results

### 3.1 Stage-dependent shifts in bacterial and fungal communities during the composting process

**Figure 1:**
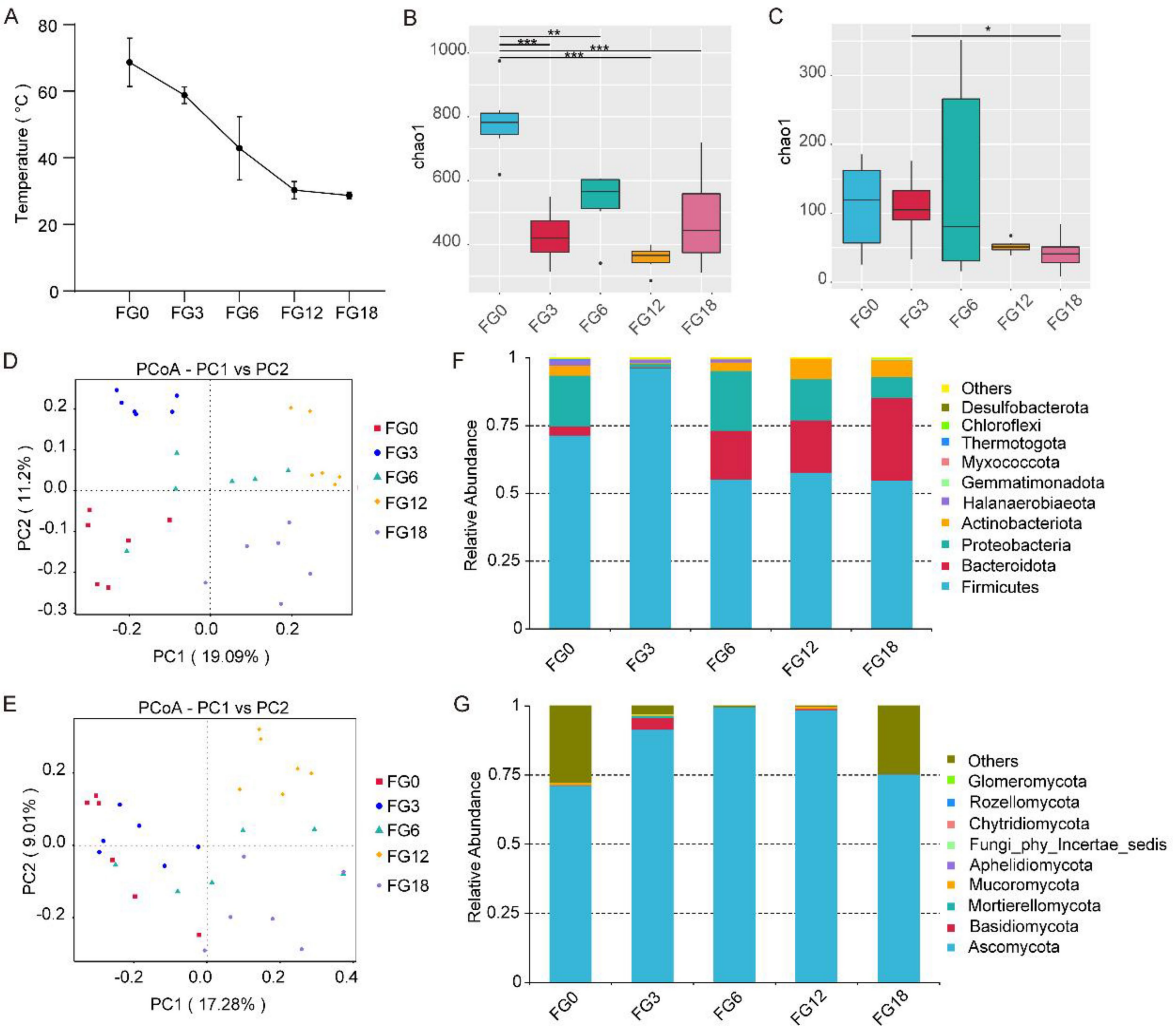
Microbial community diversity in compost samples across different periods. **(A)** Temperature changes across different compost periods. **(B)** Bacterial *α*-diversity across different compost periods. **(C)** Fungal α-diversity across different compost periods. **(D)** Principal coordinate analysis (PCoA) of bacterial community based on unweighted UniFrac distances. **(E)** PCoA of fungal community based on unweighted UniFrac distances. **(F)** Bacterial community composition at the phylum level. **(G)** Fungal community composition at the phylum level. Asterisks indicated that the *p* value is less than 0.05 (*p < 0.05; **p < 0.01; ***p < 0.001). *p* values were calculated using one-way ANOVA followed by Tukey’s *post hoc* test. Data are means ± SD.

Compost samples collected on days 0 (FG0), 3 (FG3), 6 (FG6), 12 (FG12), and 18 (FG18), covering the late thermophilic phase and the entire cooling phase of the composting process. During this period, the pile temperature gradually decreased from 68.7 °C to ambient temperature 28.7 °C (Figure 1A). To investigate microbial succession during composting, high-throughput sequencing was employed. For bacterial *α*-diversity (Figure 1B), FG0 exhibited significantly higher richness than FG3, FG6, FG12, and FG18. For fungi (Figure 1C), FG0 also showed the highest richness, although the differences among samples were not significant. PCoA analysis revealed clear separation of both bacterial and fungal communities among the five composting stages (Figures 1D,E).

At the phylum level, all samples were dominated by Firmicutes, Bacteroidota, Proteobacteria and Actinobacteriota, with Firmicutes being the most abundant taxon across all stages and peaking in FG3 (relative abundance up to 96.2%). Bacteroidota showed increased abundance during the late fermentation phase (Figure 1F). For fungi, Ascomycota was the most abundant phylum, with a relative abundance ranging from 71.3 to 99.7%, followed by Basidiomycota and Mucoromycota (Figure 1G). Overall, these results demonstrated clear, stage-dependent microbial succession during composting, characterized by the dominance of Firmicutes and Ascomycota throughout the process.

### 3.2 Distinct successional patterns of bacterial and fungal communities during composting

**Figure 2:**
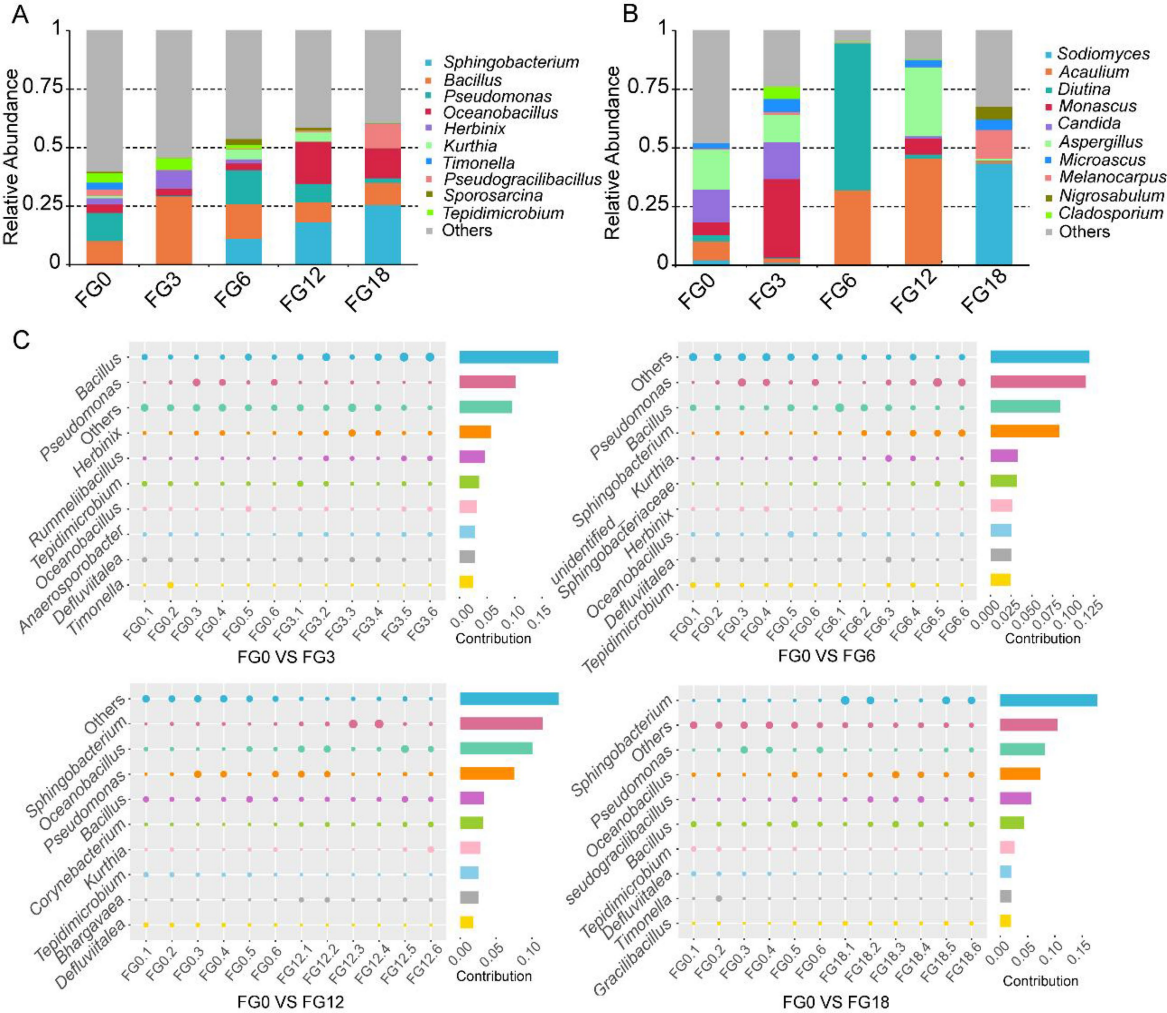
Microbial compositional differences in compost samples across different periods. **(A)** Bacterial community composition at the genus level. **(B)** Fungal community composition at the genus level. **(C)** Bacterial community differences between FG0 and other samples at the genus level and their contribution.

For bacteria (Figure 2A), the most abundant genera included *Sphingobacterium, Bacillus, Pseudomonas* and *Oceanobacillus. Bacillus* was highly abundant in all samples, with its relative abundance peaking in the FG3. In contrast, *Oceanobacillus* and *Sphingobacterium* became predominant in the FG12 and FG18. For fungi (Figure 2B), the community composition varied substantially across the five sampling times, with no single genus consistently dominating the process. For bacteria (Figure 2C), *Pseudomonas* and *Bacillus* contributed most to the differences between FG0 and other groups. For fungi (Supplementary Figure S1), *Aspergillus, Candida*, and *Thermomyces* were the key contributors to the compositional differences between FG0 and the other groups. Taken together, these results reveal that *Bacillus* drives early thermophilic activity, while fungal genera exhibit more variable patterns throughout the composting process.

### 3.3 Functional shifts of microbial communities during composting

**Figure 3:**
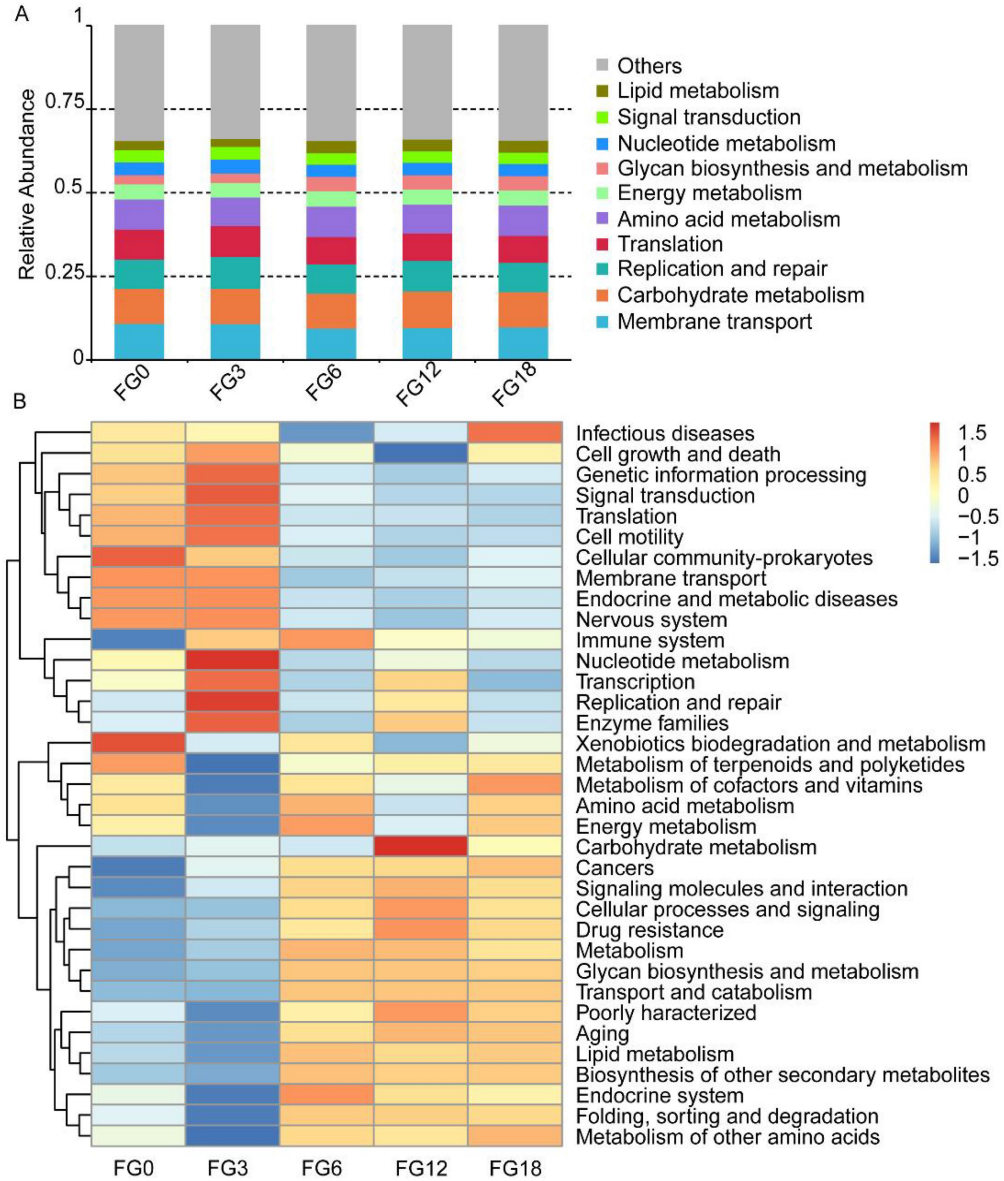
The predicted microbial functions in compost samples across different periods. **(A)** Relative abundance of the top 10 predicted functions of the bacterial community. **(B)** Heatmap illustrating the variations in predicted KEGG profiles across samples.

To investigate the functional differences of microbial communities among compost samples, Tax4Fun and FunGuild analysis were used for bacterial and fungal functional annotation, respectively. Predicted functional profiles for bacteria (Supplementary Figure S2) suggested that FG0 and FG3 groups clustered closely together and were separated from other groups (FG6, FG12, and FG18). The dominant predicted functions across samples included membrane transport, carbohydrate metabolism, replication and repair, translation, and amino acid metabolism (Figure 3A). Specifically, the bacterial community in FG0 was primarily associated with xenobiotics biodegradation and metabolism, whereas FG3 was enriched in functions related to genetic information processing (e.g., nucleotide metabolism, transcription, replication, and repair). In contrast, the FG6, FG12, and FG18 were characterized by core metabolic processes, including carbohydrate metabolism and the metabolism of glycans and other amino acids (Figure 3B). For fungi, samples did not separate clearly in the PCA analysis, indicating limited functional divergence among fungal communities across composting (Supplementary Figures S3, S4). Collectively, these predicted function results indicate that bacterial communities undergo distinct functional transitions during composting.

### 3.4 Bacterial isolation and screening for enzyme-producing strains

**Figure 4:**
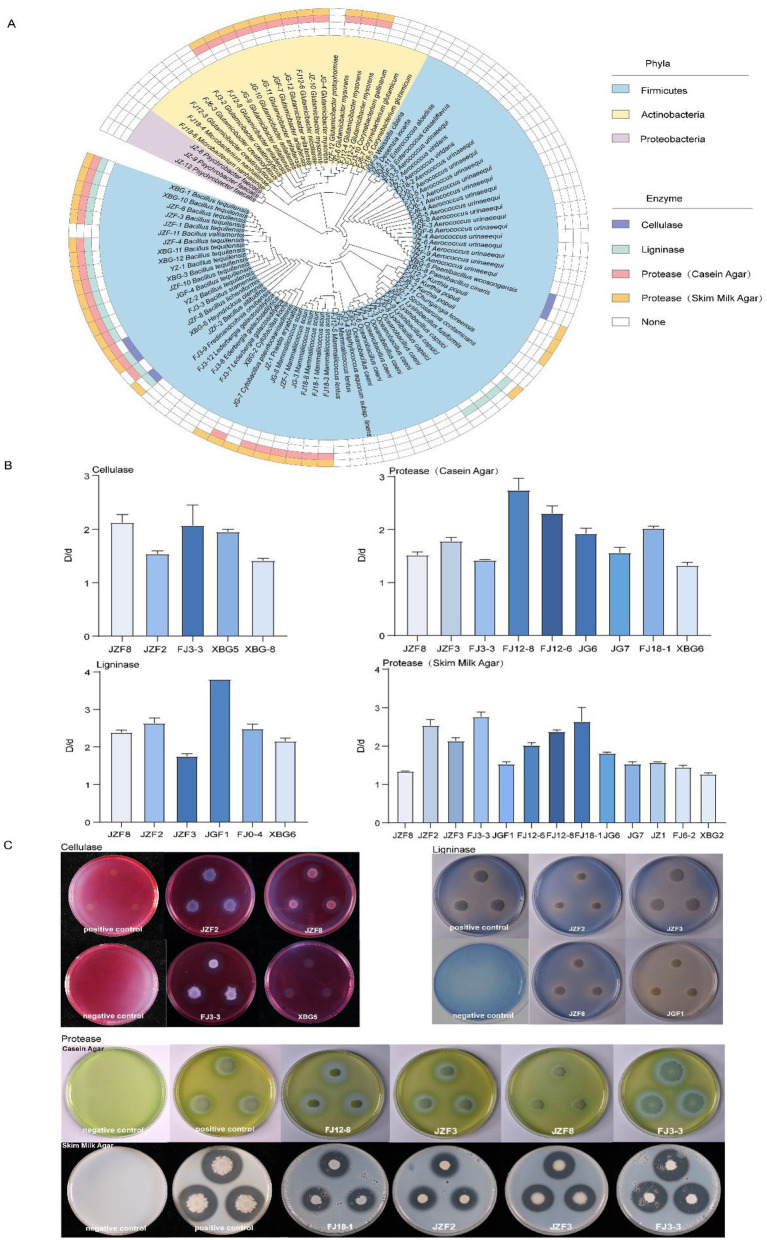
Isolation of bacterial strains and screening for enzyme production. **(A)** Phylogenetic tree of all bacterial isolates based on 16S rRNA gene sequences. **(B)** The cellulase, ligninase, and protease producing capacities of representative strains. **(C)** Plates showing cellulase, ligninase, protease activities of representative strains, positive control (a known enzyme-producing strain) and negative control (medium only). Data are means ± SD.

Based on the above microbial community analysis, the composting process substantially altered the bacterial composition and predicted functional profiles, whereas fungal communities exhibited relatively limited variation. Therefore, bacterial isolation was conducted, yielding a total of 97 strains. These isolates belonged to 3 phyla, 3 classes, 7 orders, 12 families, 23 genera and 38 species. The three phyla were Firmicutes (73 strains), Actinobacteria (21 strains) and Proteobacteria (3 strains) (Figure 4A), accounting for 75.26, 21.65, and 3.09%, respectively. At the genus level, *Aerococcus, Bacillus, Glutamicibacter, Mammaliicoccus* and *Oceanobacillus* were the five most abundant genera, accounting for 18.56, 17.53, 16.49, 8.25 and 6.19% of the isolates, respectively.

These isolated strains were further evaluated for cellulase, ligninase and protease production. Five species (five strains) exhibited cellulase activity, six species (20 strains) displayed ligninase activity, nine species (35 strains) produced protease on casein medium, and 13 species (41 strains) produced protease on skim-milk medium (Figures 4A–C; Supplementary Table S1). Among them, *Bacillus licheniformis* JZF8, *Bacillus altitudinis* JZF2, *Bacillus tequilensis* JZF3, and *Bacillus siamensis* FJ3-3 demonstrated the strongest enzymatic capabilities, each producing at least three types of functional enzymes (Supplementary Table S1).

### 3.5 Multifunctional potential of *Bacillus tequilensis* JZF3 in disease suppression and straw degradation

**Figure 5:**
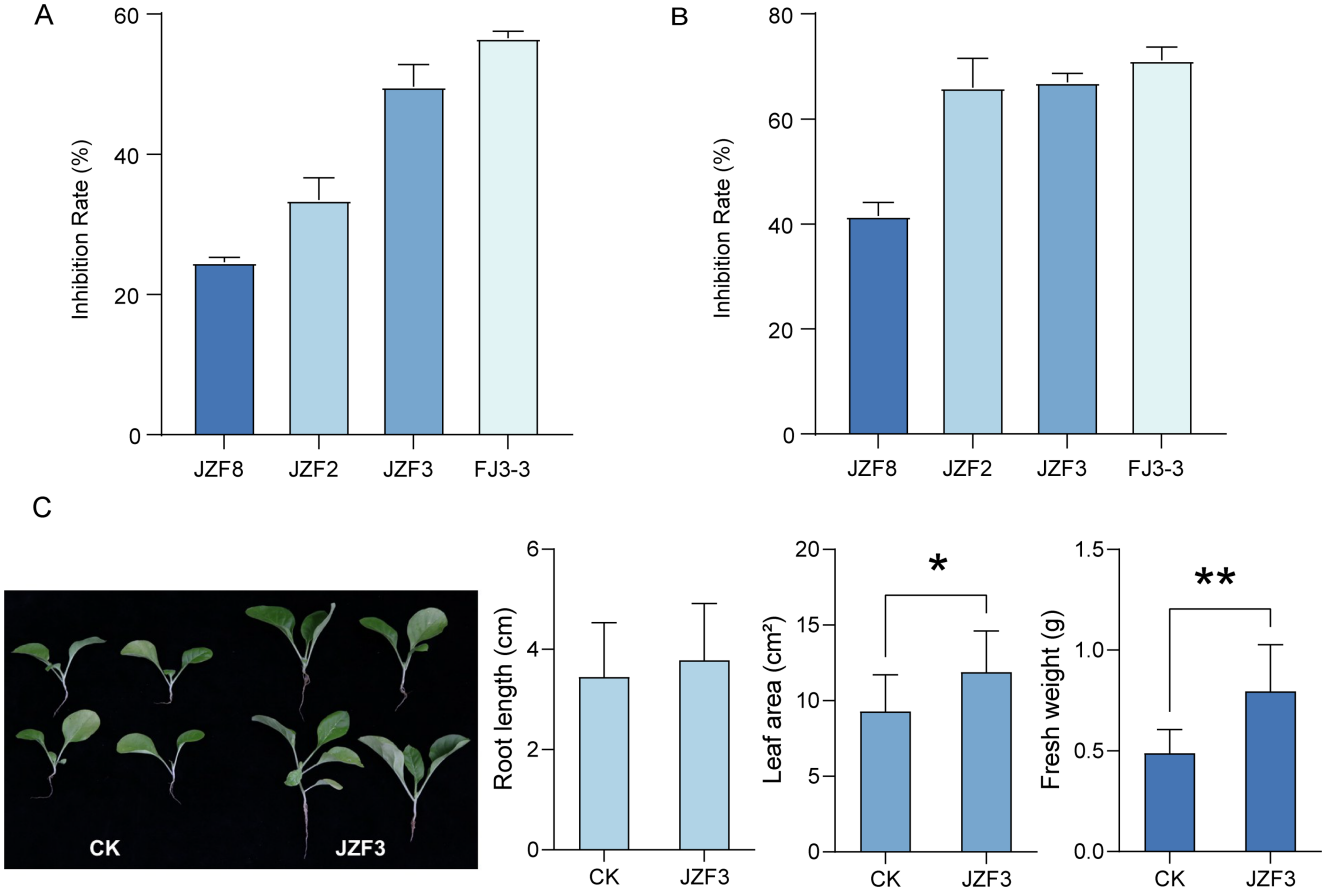
Pathogen inhibition rate and plant growth promotion of the four *Bacillus* strains. **(A)** Inhibitory rate of the four *Bacillus* strains against *Phytophthora parasitica*. **(B)** Inhibitory rate of the four *Bacillus* strains against *Alternaria alternata*. **(C)** The promoting capacity of straw degradation metabolites produced by *B. tequilensis* JZF3. Asterisk indicates that the *p* value of the two-tailed Student’s *t*-test is less than 0.05 (*p < 0.05, **p < 0.01), following assessment of data normality and variance homogeneity. Data are means ± SD.

The above four strains with strong enzymatic activities were further evaluated for their antagonistic effects against the tobacco pathogens *Phytophthora parasitica* and *Alternaria alternata*. We found that all four strains exhibited inhibitory activity against both pathogens. Among them, *B. siamensis* FJ3-3 and *B. tequilensis* JZF3 showed the strongest effects, with inhibition rates of 71.2 and 56.5% against *P. parasitica*, and 67.0 and 49.7% against *A. alternata*, respectively (Figures 5A,B; Supplementary Table S2). Notably, *B. tequilensis* accounted for 13 out of the 97 bacterial isolates, making it as the most abundant culturable *Bacillus* species in the compost community (Figure 4A). Therefore, *B. tequilensis* JZF3 was selected for further straw degradation and tobacco pot experiments. We found that strain JZF3 was able to directly degrade rice straw without any chemical pretreatment, achieving a 22.5% degradation rate after 7 days of cultivation. Considering that nutrient-rich degradation products can improve plant growth, the degradation products generated by JZF3 were then applied to tobacco seedlings. Compared with the control, treated plants showed a significant increase of 28% in leaf area (*p* < 0.05) and 63% in fresh weight (*p* < 0.05) (Figure 5C). These findings indicate that the degradation products of JZF3 can effectively promote tobacco growth, highlighting its potential as a promising agent for aerobic composting and plant growth promotion.

## 4 Discussion

Composting is a green and environmentally friendly method for treating organic waste. In this process, microorganisms play a core role in the degradation and transformation of organic matter. The microbial composition dynamics directly determine the efficiency of composting and the agricultural value of the final product (Kong et al., 2020). In this study, distiller’s grains, oil cake and cattle manure were used as aerobic composting materials. The bacterial and fungal communities were analyzed and strains with efficient lignin and cellulose degradation activities were screened.

We observed that the *α*-diversity of both bacteria and fungi peaked in FG0 sample. This finding aligns with previous composting studies, which also reported relatively high bacterial diversity during the thermophilic phase (Bellò et al., 2020; Villar et al., 2016). However, Liu et al. (2024) observed a continuous increase in microbial richness and diversity throughout the composting process using corn cobs of different particle sizes. This differs from the results of this study and may be related to the fact that the temperature increase phase was not monitored in this study. Microbial *β*-diversity analysis revealed significant variations in microbial communities across different composting periods (Figures 1D,E), indicating a notable microbial community shift across different composting periods. This result is consistent with studies on compost microbial succession by Pane et al. (2020). It should be noted that key physicochemical parameters such as moisture content, pH, and aeration during composting were not systematically measured in this study. Future work should incorporate such variables to better elucidate their correlation with microbial succession.

Firmicutes are capable of forming heat-resistant endospores, enabling them to adapt effectively to the elevated temperatures during the thermophilic phase of composting (Kong et al., 2020). This study found that Firmicutes dominated during composting (Figures 1F,G), which agrees with previous studies showing that Firmicutes, Bacteroidota, and Proteobacteria play a major role in the composting process (He et al., 2022; Antunes et al., 2016). At the genus level, *Bacillus* was the predominant taxon during composting (Figure 2A). This predominance can be attributed to several key adaptive traits. First, *Bacillus* species produce heat-resistant endospores that allow survival under thermophilic conditions and facilitate rapid germination when conditions are favorable (Kong et al., 2020; Rastogi et al., 2010). Second, many *Bacillus* species produce antimicrobial substance, which may inhibits competing microorganisms, further strengthening its ecological advantage during composting (Pliego et al., 2011). Moreover, their metabolic versatility supports the degradation of complex organic substrates like lignocellulose, allowing them to utilize diverse carbon and nitrogen sources throughout the composting process (Awasthi et al., 2020; Mei et al., 2020). Furthermore, functional prediction analysis indicated that the predicted functions of the bacterial community undergo significant succession during composting, shifting from biodegradation to core metabolic processes (Zhong et al., 2020), including carbohydrate metabolism and the metabolism of glycans and other amino acids (Figure 3B). Previous reports have indicated that carbohydrate metabolism and amino acid metabolism facilitate microbial growth and accelerate the degradation of lignocellulose (Liang et al., 2020). It should be noted that both Tax4Fun and FUNGuild infer functional profiles based on taxonomic assignments and available reference databases. Therefore, the predicted functions represent potential metabolic capabilities rather than direct evidence of *in situ* metabolic activities. Experimental validation using metagenomic, metatranscriptomic, or biochemical approaches would be required to confirm these predicted functions.

A total of 97 strains were obtained (Figure 4A), of which four *Bacillus* isolates showed the strongest enzymatic activities (Supplementary Table S1). This observation is consistent with prior reports identifying *Bacillus* as a dominant genus during the composting process (Thai et al., 2022), particularly in the degradation of lignocellulosic material (Awasthi et al., 2020). Among the *Bacillus* isolates, *B. tequilensis* JZF3 has the abilities to produce ligninase and protease, suppress phytopathogens and degrade straw (Figures 4B,C and Figures 5A,B). In field applications, such degrading metabolites could be delivered via biofertilizers or compost extracts. While straw degradation was evaluated using a weight-loss method in this study, this approach does not allow differentiation among the degradation of cellulose, hemicellulose, and lignin. Future work incorporating compositional and structural analyses would provide more detailed insights into the degradation mechanisms. It is worth noting that *B. tequilensis* has been reported to secrete lignocellulase and proteases, and its fermentation broth can promote banana plant growth (Deng et al., 2025). However, most studies on *Bacillus* focus on single functions such as lignocellulose degradation (Mei et al., 2020; Zhu et al., 2017) or biocontrol against plant pathogens (Pliego et al., 2011). In contrast, *B. tequilensis* JZF3 strain isolated in this work exhibits a unique multifunctional trait, expanding its application potential in synergizing agricultural waste recycling with disease control. Based on previous studies on *Bacillus* species, the observed inhibition may potentially be associated with the production of antimicrobial metabolites or competitive interactions for nutrients and space (Chen et al., 2025; Chen et al., 2018). Further studies incorporating biochemical and molecular analyses are required to elucidate the specific mechanisms involved.

## 5 Conclusion

In summary, this study revealed the stage-dependent succession of microbial communities during aerobic composting. *B. tequilensis* was identified as a dominant culturable species within the compost microbiota. Specifically, the strain *B. tequilensis* JZF3 showed multiple functional attributes, including the ability to degrade rice straw, strong antagonistic activity against tobacco pathogens, and plant growth-promoting effects mediated by its fermentation products. Our findings highlight the potential of *B. tequilensis* JZF3 as a promising multifunctional microbial inoculant for synergistically improving agricultural waste utilization and tobacco disease control.

## Data Availability

The datasets presented in this study can be found in online repositories. The names of the repository/repositories and accession number(s) can be found at: https://ngdc.cncb.ac.cn/gsa/, CRA034157 and CRA034185.

## Footnotes

1
https://www.ezbiocloud.net/

2
https://itol.embl.de/
