# Supplementary material for: The composting microbiome and a multifunctional Bacillus tequilensis JZF3 with straw degradation and pathogen inhibition
Source: Front Microbiol. 2026 Mar 23;17:1768200. doi: 10.3389/fmicb.2026.1768200 (PMC13050860; doi:10.3389/fmicb.2026.1768200)
Supplement: Supplementary file 1 [file Table_1.docx]

| **Supplementary**  **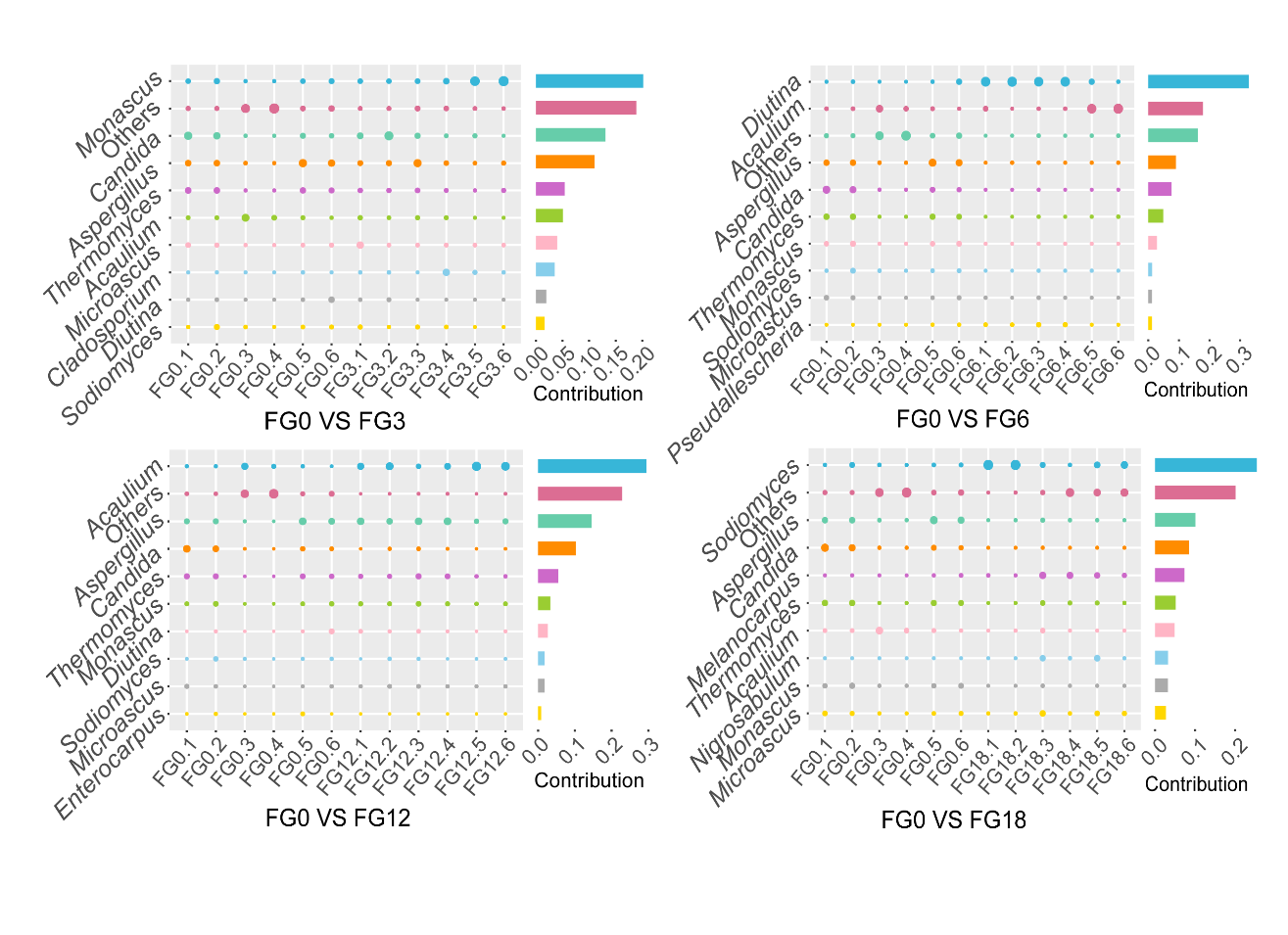**  **Figure S1 Fungal community differences between FG0 and other samples at the genus level and their contribution**  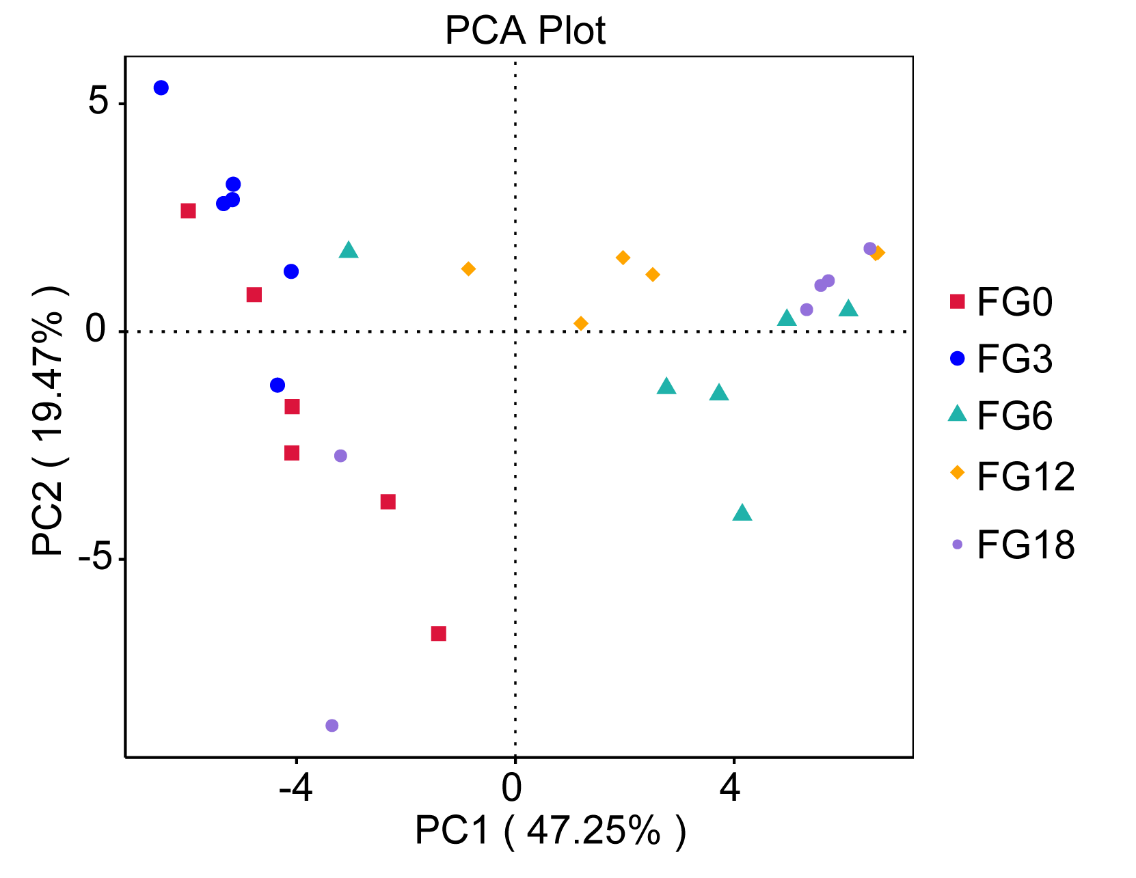  **Figure S2 PCA dimensionality reduction analysis of bacterial communities in compost samples across different periods**  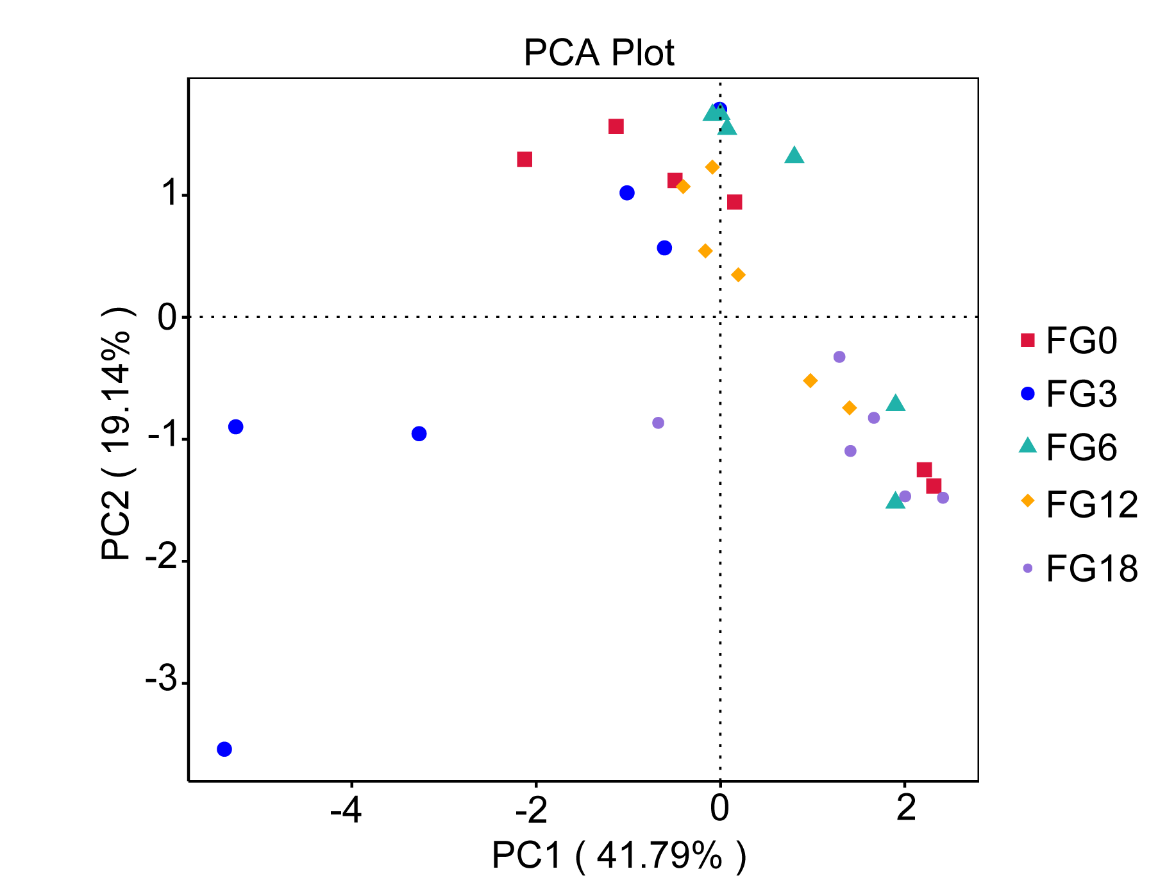  **Figure S3 PCA dimensionality reduction analysis of fungal communities in compost samples across different periods**  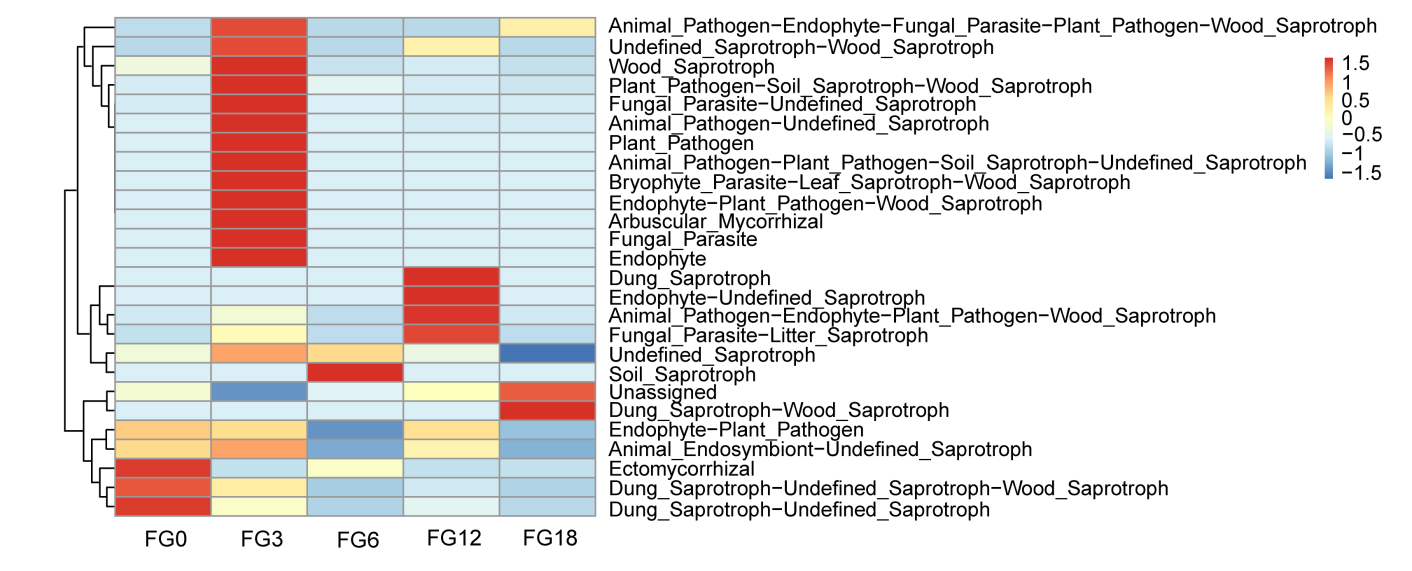 | | | | | | |
| --- | --- | --- | --- | --- | --- | --- |
| **Figure S4 Specific functional annotations of the fungal community in compost samples across different periods**  **Table S1** **The cellulase, ligninase and protease producing strains.** D, Diameter of hydrolysis circle (cm); d, Diameter of colony (cm). | | | | | | |
| Strain number | Strain | **D/d** | | | | |
|  |  | Cellulase | Ligninase | | Protease  （Casein Agar） | Protease  (Skim Milk Agar) |
| JZF8 | *Bacillus licheniformis* | 2.12 | 2.38 | | 1.52 | 1.33 |
| JZF2 | *Bacillus altitudinis* | 1.54 | 2.63 | | \ | 2.53 |
| JZF3 | *Bacillus tequilensis* | \ | 1.74 | | 1.78 | 2.13 |
| FJ3-3 | *Bacillus siamensis* | 2.07 | \ | | 1.42 | 2.76 |
| XBG5 | *Paenibacillus woosongensis* | 1.95 | \ | | \ | \ |
| XBG8 | *Paenibacillus cineris* | 1.41 | \ | | \ | \ |
| XBG6 | *Heyndrickxia oleronia* | \ | 2.15 | | 1.32 | \ |
| JGF1 | *Lysinibacillus fusiformis* | \ | 3.8 | | \ | 1.52 |
| FJ0-4 | *Lysinibacillus capsici* | \ | 2.48 | | \ | \ |
| FJ12-6 | *Glutamicibacter nicotianae* | \ | \ | | 2.31 | 2.01 |
| FJ12-8 | *Glutamicibacter arilaitensis* | \ | \ | | 2.74 | 2.37 |
| FJ18-1 | *Mammaliicoccus sciuri* | \ | \ | | 2.02 | 2.63 |
| JG6 | *Glutamicibacter mysorens* | \ | \ | | 1.92 | 1.81 |
| JG7 | *Cytobacillus pseudoceanisediminis* | \ | \ | | 1.56 | 1.53 |
| JZ1 | *Priestia aryabhattai* | \ | \ | | \ | 1.57 |
| FJ6-2 | *Kurthia populi* | \ | \ | | \ | 1.44 |
| XBG2 | *Cytobacillus kochii* | \ | | \ | \ | 1.27 |

**Table S2 The inhibition rate of the strain.**

| Strain number | Strain | Inhibitory Rates (%) | |
| --- | --- | --- | --- |
|  |  | *Phytophthora parasitica* | *Alternaria alternata* |
| JZF2 | *Bacillus altitudinis* | 66.0% | 33.5% |
| JZF3 | *Bacillus tequilensis* | 67.0% | 49.7% |
| JZF8 | *Bacillus licheniformis* | 41.5% | 24.6% |
| FJ3-3 | *Bacillus siamensis* | 71.2% | 56.5% |
